# Time to Treatment Initiation and Survival in Adult Localized High-Grade Bone Sarcoma

**DOI:** 10.1155/2020/2984043

**Published:** 2020-05-04

**Authors:** Joshua M. Lawrenz, Joseph Featherall, Gannon L. Curtis, Jaiben George, Yuxuan Jin, Peter M. Anderson, Dale R. Shepard, John D. Reith, Brian P. Rubin, Lukas M. Nystrom, Nathan W. Mesko

**Affiliations:** ^1^Vanderbilt University Medical Center, Nashville, TN 37232, USA; ^2^University of Utah Hospital, Salt Lake City, UT 84132, USA; ^3^University Health Center, Wayne State University, Detroit, MI 48201, USA; ^4^AIIMS Hospital, New Delhi, India; ^5^Cleveland Clinic, Cleveland, OH 44195, USA

## Abstract

**Objective:**

Few studies have evaluated the prognostic implication of the length of time from diagnosis to treatment initiation in bone sarcoma. The purpose of this study is to determine if time to treatment initiation (TTI) influences overall survival in adults diagnosed with primary bone sarcoma.

**Methods:**

A retrospective analysis of the National Cancer Database identified 2,122 patients who met inclusion criteria with localized, high-grade bone sarcoma diagnosed between 2004 and 2012. TTI was defined as length of time in days from diagnosis to initiation of treatment. Patient, disease-specific, and healthcare-related factors were also assessed for their association with overall survival. Kruskal-Wallis analysis was utilized for univariate analysis, and Cox regression modeling identified covariates associated with overall survival.

**Results:**

Any 10-day increase in TTI was not associated with decreased overall survival (hazard ratio (HR) = 1.00; *P*=0.72). No differences in survival were detected at 1 year, 5 years, and 10 years, when comparing patients with TTI = 14, 30, 60, 90, and 150 days. Decreased survival was significantly associated (*P* < 0.05) with patient ages of 51–70 years (HR = 1.66; *P*=0.004) and > 71 years (HR = 2.89; *P* < 0.001), Charlson/Deyo score ≥2 (HR = 2.02; *P* < 0.001), pelvic tumor site (HR = 1.58; *P* < 0.001), tumor size >8 cm (HR = 1.52; *P* < 0.001), radiation (HR = 1.81; *P* < 0.001) as index treatment, and residing a distance of 51–100 miles from the treatment center (HR = 1.30; *P*=0.012). Increased survival was significantly associated (*P* < 0.05) with chordoma (HR = 0.27; *P*=0.010), chondrosarcoma (HR = 0.75; *P*=0.002), treatment at an academic center (HR = 0.64; *P*=0.039), and a private (HR = 0.67; *P*=0.006) or Medicare (HR = 0.71; *P*=0.043) insurer. A transition in care was not associated with a survival disadvantage (HR = 0.90; *P*=0.14).

**Conclusions:**

Longer TTI was not associated with decreased overall survival in localized, high-grade primary bone sarcoma in adults. This is important in counseling patients, who may delay treatment to receive a second opinion or seek referral to a higher volume sarcoma center.

## 1. Introduction

Primary bone sarcomas are rare malignancies with a national incidence in the United States of around 3,200 cases annually and a five-year relative survival between 60 and 70% in localized disease [1]. Prognosis in bone sarcoma is closely correlated with tumor grade and disease stage, which argues for earlier diagnosis and treatment [2, 3]. Time to treatment initiation (TTI), defined as the duration of time between diagnosis and the initiation of treatment, has become an important quality metric in cancer care, as the length of this time period can potentially affect patient anxiety and outcome. Registry data for breast and head and neck cancers have demonstrated an association between increased treatment wait times and decreased survival [4, 5]. It is arguable that the potential benefits of shorter TTI would apply to most, if not all, cancers, including high-grade bone sarcoma. Despite the obvious benefits of expedited treatment, other factors such as treatment at an established multidisciplinary sarcoma program are believed to positively affect prognosis but may result in a treatment delay due to coordination and transfer of care [6–8]. Thus, the inquiry as to if TTI affects prognosis in bone sarcoma is nuanced, and the rarity of the disease has led to limited data addressing this issue [9].

The National Cancer Database (NCDB) is a high-quality cancer registry that captures data from newly diagnosed cancers in the United States and is of particular value when investigating rare cancers such as bone sarcoma [10]. The NCDB has been utilized to investigate the correlation between time to treatment and survival in other cancer types in effort to reduce delays and improve outcomes [4, 5, 11]. In a recent inquiry of the effect of TTI on survival in soft tissue sarcoma, TTI was found to have minimal effect on overall survival (OS), with a delay of greater than 42 days having a trend toward decreasing survival [7]. No similar studies have been performed with the primary goal to establish this correlation in bone sarcoma.

The primary aim of this study was to determine if TTI influences OS in patients diagnosed with localized, high-grade bone sarcoma. We hypothesized that prolonged TTI would be associated with decreased survival in bone sarcoma patients. Additionally, the secondary aim was to identify patient socioeconomic, tumor-specific, and healthcare-related factors that contribute to bone sarcoma survival.

## 2. Methods

### 2.1. Database and Selection of Patients

**Figure 1:**
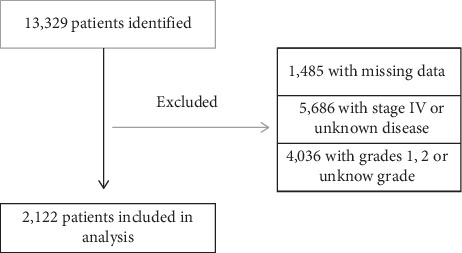
Study cohort inclusion criteria.

Following approval by our institutional review board, the NCDB was reviewed from 2004 to 2012. Created in 1989 by the American College of Surgeons (ACS) and the Commission on Cancer (CoC), the NCDB captures 70% of all new United States cancer diagnoses and collects data from over 1,500 cancer centers [12]. The methodology for reporting to the NCDB has been previously described [8]. Adult patients (≥18 years old) with bone sarcoma diagnosed between 2004 and 2012 were identified using topographical codes (C40.0-C40.3, C40.8-C41.4, C41.8, C41.9) designated by International Classification of Disease for Oncology, Third Edition [ICD-O-3]. A patient also required an ICD-O-3 histology code consistent with a bone sarcoma to be included. These codes identified a total of 13,329 patients with a bone sarcoma. Patients were excluded for the following reasons: (1) lack of follow-up or essential data (*n* = 1,485), (2) American Joint Committee on Cancer (AJCC) Stage IV or unknown stage disease (*n* = 5,686), and (3) well differentiated (grade 1), moderately differentiated (grade 2), or unknown grade (*n* = 4,036). Thus, 2,122 adult patients with localized, high-grade disease were included in the final analysis. Given the significant impact tumor grade and disease stage have been shown to have on survival outcome [9, 13, 14], this cohort was intentionally limited to patients with high grade, localized disease. The inclusion criteria can be found in Figure 1.

### 2.2. Outcome Measures

The primary objective of this study was to evaluate the association between TTI and OS in patients with localized, high-grade bone sarcoma. TTI was defined as the time in days between confirmed tissue diagnosis and initiation of any definitive treatment course (surgical resection, systemic chemotherapy, and radiation therapy). Diagnostic or palliative procedures do not qualify as treatment initiation. OS was defined as the time in months from treatment initiation until death or the patient's last follow-up visit. Patient, healthcare, and tumor characteristics (Table 1) were also collected to investigate their associations with patient OS. Patient demographics included age, gender, race, Charlson/Deyo Score (CDS) (0, 1, or >2), and annual income. It is important to note that annual income is not patient derived data, but rather the mean income reported in the patient's zip code. Tumor factors included histology, primary site, size, grade, clinical stage, and initial definitive treatment modality. Healthcare system factors included treating facility type, insurance provider, distance from the patient's residence to the treating facility, and presence of a transition in care. Patients who received a diagnosis at one facility and had initial treatment commencement at another facility were considered to have a transition in care. Facility type was divided into community cancer programs, comprehensive cancer centers, academic centers, integrated network cancer programs, and other. Community cancer programs are defined as having 100–500 new cancer cases a year, whereas comprehensive cancer programs are defined as diagnosing >500 new cancer cases a year. “New cancer cases” are defined as all histologic diagnoses, not exclusively sarcoma. Community programs offer both diagnostic and treatment services, although what specific treatment services offered for rare malignancies such as sarcoma are unknown. Integrated network cancer programs usually have a “unified cancer committee” and consist of “multiple facilities providing comprehensive services” [15]. Academic institutions are defined with the same patient volume definition as a comprehensive cancer center but also have a noted resident/medical education program.

### 2.3. Statistical Analysis

The number of patients and frequencies for all independent categorical variables were reported. Median TTI was reported given the nonparametric dataset and was compared across different levels of the same categorical variable by using Kruskal-Wallis tests. The relationship between OS and TTI, along with other important secondary covariates such as age, gender, race, and treatment modality were examined with Cox regression modeling. Hazard ratios (HR) and 95% confidence intervals (CI) were determined for each variable. The TTI variable was entered into the full Cox regression by using four-knot restricted cubic splines to allow for a nonlinear relationship between TTI and the survival outcome [16]. However, the spline effect was not significant. Given the nonsignificant and nonlinear relationship of TTI with survival in all TTI cohorts, cubic spline modeling of HR according to TTI as a continuous variable was not performed. After specifying different TTI values (TTI = 14, 30, 60, 90, and 150 days) and by setting the covariates to their reference levels, the 1-year, 5-year, and 10-year survival probabilities were determined and associated survival curves were plotted. Statistical analyses were completed with SAS software (Version 9.4; Cary, NC). The multivariable cox regression model was built using rms package in R software (Version 3.4; Vienna, Austria). All tests were two-sided, with an alpha level of 0.05. *P* values less than 0.05 were considered significant.

## 3. Results

### 3.1. TTI and Survival

**Figure 2:**
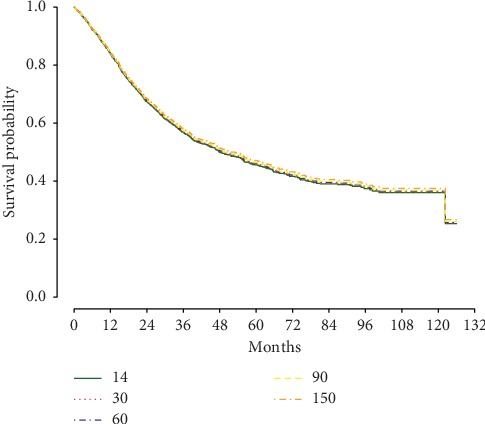
Survival curves using different values for time to treatment initiation. This graph demonstrates the near-identical Kaplan–Meier survival curves when comparing patients with a time to treatment initiation of 14, 30, 60, 90, and 150 days (HR = 1.00; *P*=0.72).

Overall survival probabilities demonstrated minimal differences at 1 year, 5 years, and 10 years at TTI = 14, 30, 60, 90, and 150 days (Table 2). Similarly, adjusted survival curves generated by Cox regression modeling were near identical out to 10 years (HR = 1.00; *P*=0.72) (Figure 2).

### 3.2. Factors That Influence Survival

**Figure 3:**
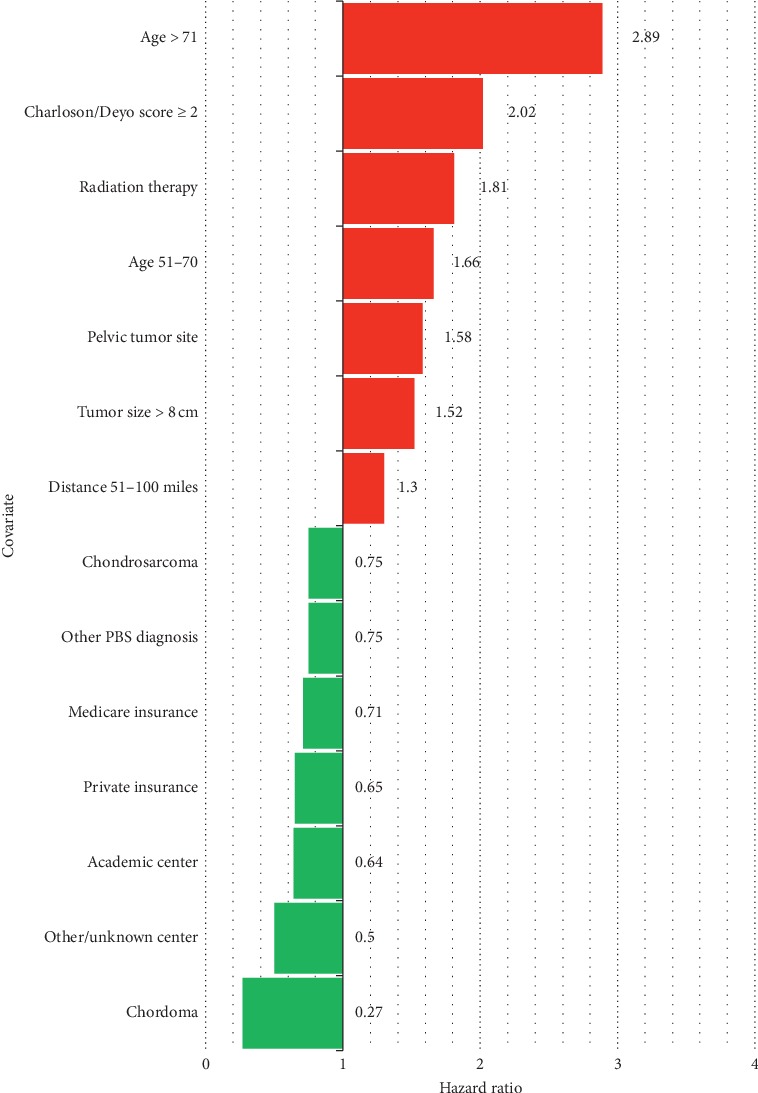
Comparison of relative association between covariates and survival. Only covariates with statistically significant higher (red) or lower (green) HR are shown.

Univariate analysis revealed significant differences seen in regard to the relationship of TTI with several secondary variables (Table 3). Multivariable analysis also identified several secondary patient, tumor, treatment, and healthcare system related factors associated with mortality (Table 4). Those that were statistically significant are highlighted in Figure 3. Patient factors such as age between 51 and 70 (HR = 1.66; *P* = 0.004) and age of 71+ (HR = 2.89; *P* < 0.001) and patients with a Charlson/Deyo score ≥2 (HR = 2.02; *P* < 0.001) were associated with decreased survival, whereas sex, race, and income were not associated with survival. A diagnosis of chondrosarcoma (HR = 0.75; *P* = 0.002), chordoma (HR = 0.27; *P* = 0.01), or other bone sarcoma not including Ewing's sarcoma (HR = 0.75; *P* = 0.022) all were associated with increased survival when compared to osteosarcoma, whereas tumors located in the pelvis (HR = 1.58; *P* < 0.001) and tumors greater than 8 cm in size (HR = 1.52; *P* < 0.001) were associated with decreased survival. Being a distance between 51 and 100 miles from the treatment center (HR = 1.30; *P* = 0.012) compared to being less than 21 miles away was associated with decreased survival, though being greater than 100 miles away had no effect. Any year of diagnosis between 2005 and 2012 compared to 2004 did not influence prognosis. Patients treated at an academic center (HR = 0.64; *P* = 0.039) or other noncategorized center (HR = 0.50; *P* = 0.006) compared to a community cancer program had an association with increased survival. Patients with private insurance (HR = 0.65; *P* = 0.004) or Medicare insurance (HR = 0.71; *P* = 0.043) had an association with increased survival. Having a transition in care after diagnosis to another center for treatment did not influence survival outcome (HR = 0.90; *P* = 0.14). First-line treatment of radiation therapy (HR = 1.81; *P* < 0.001) when compared to surgery as first treatment had an association with decreased survival. Tumor grade and clinical stage did not demonstrate association with survival, as to be expected in a cohort of only high grade, localized bone sarcomas.

## 4. Discussion

These data demonstrate that all cause survival probability at one, five, and ten years after diagnosis was no different when comparing patients with a TTI ranging from 0 to 150 days (five months). Factors found to correlate with survival included patient age, comorbidity index, histologic subtype, primary tumor location and size, initial treatment type, type of insurance, treating facility type, and distance of home residence from the treating facility.

Prior data associating treatment delay with survival outcome in sarcoma is limited, with only a single study that compared a treatment delay of less than or greater than three weeks [9]. The authors concluded no difference on survival outcome in their cohort of extremity osteosarcomas. In both breast and head and neck cancers, recent registry data have shown a correlation between increased treatment wait times and decreased survival [4, 5]. Nevertheless, given the findings of the present study and previous work in soft tissue sarcoma [7], it remains unclear as to why TTI has little prognostic implication in sarcoma.

Far more studied is the association between time to diagnosis and survival, as delay in diagnosis is the most common reason for litigation related to the treatment of extremity sarcoma [17]. The traditional legal argument is that the increased time allows for a cancer to grow and spread, leading to worse prognosis. Prior studies have evaluated the length of time prior to diagnosis (or duration of symptoms) in bone sarcoma and have demonstrated no significant correlation with survival [13, 14]. This information is useful when counseling patients who exhibit remorse or anxiety for not presenting to a physician sooner. Considering the lack of correlation between longer duration of symptoms and worsened survival, it is perhaps not surprising that TTI (which is typically a much shorter time period than time to diagnosis, 3 weeks [8] vs. 16 weeks [13]), similarly found no difference. Factors rooted in tumor biology, outside the control of the treating team, are likely a powerful confounding factor in understanding the natural history of primary bone sarcoma.

In a 2019 analysis utilizing the NCDB population, Lawrenz et al. identified patient and disease-specific factors that correlated with TTI in over 13,000 patients with bone sarcoma, highlighting transitions in care from one treating facility to another as being responsible for the greatest increases in TTI [8]. Other factors associated with longer TTI included uninsured or government insurer status, nonwhite race, pelvic tumor location, and treatment at an academic center. A secondary aim of this study was to identify patient, tumor, and healthcare system factors associated with survival. Understanding the overwhelming influence tumor grade and disease stage have been shown to have on prognosis [9, 13, 14], this cohort was intentionally limited to patients with high grade, localized disease. Similar to prior work, this data reiterates that increased patient age (>51 years), increased tumor size (>8 cm), and pelvic tumor location are correlated with decreased survival, and a diagnosis of chondrosarcoma or chordoma are correlated with increased survival [13]. It was not surprising to learn that patients who underwent radiation therapy as first treatment (86 patients, 4.1%) had an associated worse prognosis compared to patients who underwent surgery or systemic therapy first, as we suspect this cohort was likely biased toward unresectable tumors or patients undergoing palliation. Furthermore, patients who lived a distance of 51–100 miles from the treatment center compared to those who lived <21 miles away had an increased risk of death, despite having a shorter median TTI (23 days vs. 25 days, respectively). To no surprise, insured status (private insurer or Medicare insurer) when compared to being uninsured was found to be associated with increased survival, similar to recently reported trends seen in prostate, lung, and colorectal cancer [18]. Furthermore, our data supported the previously noted correlation between receiving care at a high-volume facility and improved survival outcome [7, 19, 20]. As well, a transition in care, which previously has been shown to have the greatest correlation with longer TTI [8], was not associated with a survival disadvantage. This supports the concept of referral to a sarcoma referral center with a multidisciplinary treatment team, despite the likely delay in treatment initiation.

This study has several limitations. A retrospective analysis utilizing multivariable regression only allows for determination of correlation between factors and an outcome, not causation. We recognize there are factors not included in our analysis which remain unaccounted for or uncontrolled. To this end, we sought to reduce the potential confounding effect of severity of disease and its known strong correlation with prognosis by restricting this cohort to only patients with localized, high-grade disease. Despite this, we recognize this cohort of bone sarcomas consists of multiple histology types, in which there can be differences amongst individual types on prognosis, which may blunt the effect of treatment delay in the cohort as a whole. Furthermore, national registries are prone to incomplete data reporting and even unknown data collection errors. In this dataset, there were 1,485 patients missing time to treatment data which we excluded. As well, 40% of patients were categorized as “other/unknown treatment facility type.” Given that this was not a critical factor in assessing our primary endpoint, we included these patients for the sake of increased sample size, though making conclusions regarding this specific variable more difficult to interpret. Despite this, when studying a rare disease such as sarcoma, tools such as the NCDB though imperfect provide a large cohort to investigate important questions for the purposes of data description and hypothesis generation. These limitations could be largely improved upon with a multi-institutional prospective registry effort focused on sarcoma diagnoses.

In conclusion, this analysis of the NCDB from 2004 to 2012 demonstrates TTI does not correlate with overall survival in localized, high-grade primary bone sarcoma in adults. The primary and secondary conclusions of this data suggest that factors inherent to the patient, disease process, and treating facility are likely more integral to overall prognosis, rather than the length of time from when a diagnosis is made and when treatment is initiated. This is important in counseling patients, who may delay treatment to receive a second opinion or seek referral to a higher volume sarcoma center.

## Figures and Tables

**Table 1:** Demographic data.

Factor	Total (*N* = 2,122)
Time to treatment initiation, days (IQR)	25.0 [12.0, 42.0]
Age	
18–30	654 (30.8%)
31–50	555 (26.2%)
51–70	627 (29.5%)
71+	286 (13.5%)
Sex	
Male	1,241 (58.5%)
Female	881 (41.5%)
Race	
White	1,735 (81.8%)
Black	258 (12.2%)
Other/unknown	129 (6.1%)
Charlson/Deyo score	
0	1,843 (86.9%)
1	222 (10.5%)
≥2	57 (2.7%)
Histology	
Osteosarcoma	1,217 (57.4%)
Chondrosarcoma	486 (22.9%)
Ewing's sarcoma	195 (9.2%)
Chordoma	17 (0.80%)
Other	207 (9.8%)
Facility type	
Comm. cancer prg.	38 (1.8%)
Comprehensive comm. cancer prg.	286 (13.5%)
Academic center	856 (40.3%)
Integrated network cancer prg.	62 (2.9%)
Other/unknown	880 (41.5%)
Insurance	
Uninsured	120 (5.7%)
Private insurance	1,148 (54.1%)
Medicaid	258 (12.2%)
Medicare	454 (21.4%)
Other/unknown	142 (6.7%)
Income	
<$38,000	404 (19.0%)
$38,000-$47,999	500 (23.6%)
$48,000-$62,999	563 (26.5%)
$63,000+	655 (30.9%)
Distance from facility	
<21 miles	974 (45.9%)
21–50 miles	466 (22.0%)
51–100 miles	306 (14.4%)
>100 miles	376 (17.7%)
Transition in care	
No	1,137 (53.6%)
Yes	985 (46.4%)
Year of diagnosis	
2004	160 (7.5%)
2005	213 (10.0%)
2006	226 (10.7%)
2007	202 (9.5%)
2008	244 (11.5%)
2009	273 (12.9%)
2010	274 (12.9%)
2011	267 (12.6%)
2012	263 (12.4%)
Primary tumor site	
Upper extremity	281 (13.2%)
Lower extremity	985 (46.4%)
Pelvis	344 (16.2%)
Other	512 (24.1%)
Tumor size	
≤8.0 cm	967 (45.6%)
>8.0 cm	1,155 (54.4%)
Grade	
Poorly differentiated	1,253 (59.0%)
Undifferentiated	869 (41.0%)
Clinical staging	
Stage I	239 (11.3%)
Stage II	1,782 (84.0%)
Stage III	101 (4.8%)
First-line treatment modality	
Surgery	1,029 (48.5%)
Radiation	86 (4.1%)
Systemic	994 (46.8%)
Multimodal	13 (0.61%)
Vital status	
Died	922 (43.4%)
Alive	1,200 (56.6%)

Statistics presented as median [P25, P75] or N (column %). Community cancer program: between 100 and 500 new cancer cases annually, Comprehensive community cancer program: >500 new cancer cases annually, academic center: >500 new cancer cases annually and resident/medical education, integrated network: multiple facilities providing comprehensive services with a unified cancer committee. comm., community; prg., program.

**Table 2:** 1-year, 5-year, and 10-year survival probabilities based upon time to treatment initiation.

Time	Survival probability	95% CI	
TTI = 14			
1 year	0.84	0.74	0.95
5 years	0.46	0.26	0.79
10 years	0.36	0.18	0.74
TTI = 30			
1 year	0.84	0.74	0.95
5 years	0.46	0.26	0.79
10 years	0.36	0.18	0.74
TTI = 60			
1 year	0.84	0.74	0.95
5 years	0.46	0.27	0.80
10 years	0.37	0.18	0.75
TTI = 90			
1 year	0.84	0.75	0.95
5 years	0.46	0.27	0.80
10 years	0.37	0.18	0.75
TTI = 150			
1 year	0.85	0.75	0.96
5 years	0.47	0.27	0.82
10 years	0.38	0.18	0.78

TTI: time to treatment initiation; CI: confidence interval.

**Table 3:** Univariate relationships between factors and time to treatment initiation.

Factor	N	TTI, days median [p25, p75]	*P* value
Age			*0*.*006*
18–30	654	21.0 [13.0, 366.0]	
31–50	555	28.0 [14.0, 44.0]	
51–70	627	26.0 [12.0, 45.0]	
71+	286	25.5 [7.0, 44.0]	
Sex			0.52
Male	1241	24.0 [12.0, 42.0]	
Female	881	26.0 [13.0, 43.0]	
Race			*0*.*050*
White	1735	25.0 [12.0, 42.0]	
Black	258	28.0 [22.0, 48.0]	
Other/unknown	129	20.0 [7.0, 44.0]	
Charlson/Deyo score			0.18
0	1843	25.0 [13.0, 42.0]	
1	258	22.0 [9.0, 40.0]	
≥2	57	29.0[7.0, 44.0]	
Histology			*0*.*003*
Osteosarcoma	1217	25.0 [13.0, 40.0]	
Chondrosarcoma	486	27.0 [10.0, 48.0]	
Ewing's sarcoma	195	21.0 [11.0, 34.0]	
Chordoma	17	38.0 [17.0, 77.0]	
Other	207	29.0 [15.0, 49.0]	
Facility type			*<0*.*001*
Comm. cancer prg.	38	32.5 [1.00, 48.0]	
Comprehensive comm. cancer Prg.	286	21.5 [5.0, 40.0]	
Academic center	856	27.0 [14.0, 47.0]	
Integrated network cancer program	62	29.5 [11.0, 52.0]	
Other/unknown	880	23.0 [13.0, 37.0]	
Insurance			*<0*.*001*
Uninsured	120	27.5 [15.0, 43.5]	
Private insurance	1148	23.0 [12.0, 39.0]	
Medicaid	258	26.0 [13.0, 43.0]	
Medicare	454	27.5 [10.0, 48.0]	
Other/unknown	142	35.0 [19.0, 52.0]	
Income			0.73
<$38,000	404	25.5 [12.0, 45.5]	
$38,000–$47,999	500	24.0 [12.0, 40.5]	
$48,000–$62,999	563	24.0 [11.0, 43.0]	
$63,000+	655	26.0 [14.0, 42.0]	
Distance from facility			0.069
>21 miles	974	25.0 [12.0, 42.0]	
21–50 miles	466	24.0 [13.0, 42.0]	
51–100 miles	306	23.0 [9.0, 40.0]	
>100 miles	376	28.0 [14.5, 44.0]	
Transition in care			*<0*.*001*
No	1137	20.0 [8.0, 35.0]	
Yes	985	31.0 [19.0, 49.0]	
Year of diagnosis			0.25
2004	160	25.5 [9.0, 42.0]	
2005	213	27.0 [14.0, 51.0]	
2006	226	24.0 [11.0, 39.0]	
2007	202	25.5 [13.0, 43.0]	
2008	244	25.0 [13.0, 42.0]	
2009	273	23.0 [11.0, 39.0]	
2010	274	25.5 [14.0, 41.0]	
2011	267	27.0 [14.0, 44.0]	
2012	263	25.0 [12.0, 42.0]	
Primary tumor site			*<0*.*001*
Upper extremity	281	25.0 [14.0, 42.0]	
Lower extremity	985	22.0 [12.0, 36.0]	
Pelvis	344	29.0 [15.0, 49.0]	
Other	512	28.0 [9.5, 48.0]	
Tumor size			0.48
≤8.0 cm	967	26.0 [12.0, 42.0]	
>8.0 cm	1155	25.0 [13.0, 42.0]	
Grade			0.63
Poorly differentiated	1253	25.0 [11.0, 43.0]	
Undifferentiated	869	26.0 [14.0, 42.0]	
Clinical staging			0.10
Stage I	239	28.0 [14.0, 49.0]	
Stage II	1782	24.0 [12.0, 42.0]	
Stage III	101	27.0 [11.0, 45.0]	
First-line treatment modality			*<0*.*001*
Surgery	1029	24.0 [6.0, 47.0]	
Radiation	86	34.5 [19.0, 56.0]	
Systemic	994	25.0 [15.0, 37.0]	
Multimodel	13	39.0 [26.0, 47.0]	

*P* values correspond to the Kruskal–Wallis test. Comm.: community; prg.: program; TTI: time to treatment initiation.

**Table 4:** Multivariate analysis of factors associated with survival.

Factors		Hazard ratio	95% hazard ratio CI		*P* values
Time to treatments, days	TTI—a 10-day increase from day 14	1.00	0.98	1.02	0.72
	TTI—a 10-day increase from day 30	1.00	0.98	1.02	0.72
	TTI—a 10-day increase from day 60	1.00	0.98	1.02	0.72
	TTI—a 10-day increase from day 90	1.00	0.98	1.02	0.72
	TTI—a 10-day increase from day 150	1.00	0.98	1.02	0.72
Age group, years	31–50 vs. 18–30	1.16	0.88	1.51	0.29
	51–70 vs. 18–30	1.66	1.17	2.34	*0*.*004*
	71+ vs. 18–30	2.89	1.95	4.28	*<0*.*001*
Sex	Female vs. male	0.94	0.82	1.07	0.33
Race	Black vs. white	0.94	0.75	1.17	0.59
	Other/unknown vs. white	0.98	0.73	1.31	0.88
Charlson/Deyo score	1 vs. 0	1.11	0.90	1.36	0.34
	≥2 vs. 0	2.02	1.45	2.81	*<0*.*001*
Income	$38,000–$47,999 vs. <$38,000	1.08	0.88	1.33	0.45
	$48,000–$62,999 vs. <$38,000	1.03	0.84	1.27	0.75
	$63,000+ vs. <$38,000	1.04	0.84	1.29	0.70
Insurance	Private insurance vs. uninsured	0.65	0.49	0.87	*0*.*004*
	Medicaid vs. uninsured	0.85	0.61	1.18	0.33
	Medicare vs. uninsured	0.71	0.51	0.99	*0*.*043*
	Other/unknown vs. uninsured	0.75	0.51	1.10	0.15
Facility type	Comprehensive community cancer program vs. community cancer program	0.89	0.58	1.39	0.62
	Academic center vs. community cancer program	0.64	0.41	0.98	*0*.*039*
	Integrated network cancer program vs. community cancer program	0.99	0.58	1.68	0.97
	Other/unknown vs. community cancer program	0.50	0.30	0.82	*0*.*006*
Distance from facility	21–50 miles vs. <21 miles	1.17	0.98	1.39	0.088
	51–100 miles vs. <21 miles	1.30	1.06	1.59	*0*.*012*
	>100 miles vs. <21 miles	1.08	0.88	1.33	0.44
Transition in care	Yes vs. No	0.90	0.78	1.04	0.14
Year of diagnosis	2005 vs. 2004	1.06	0.79	1.41	0.71
	2006 vs. 2004	0.84	0.63	1.13	0.26
	2007 vs. 2004	1.18	0.88	1.58	0.27
	2008 vs. 2004	0.84	0.62	1.13	0.25
	2009 vs. 2004	0.90	0.67	1.21	0.48
	2010 vs. 2004	1.00	0.74	1.35	0.99
	2011 vs. 2004	1.22	0.90	1.65	0.19
	2012 vs. 2004	1.06	0.76	1.47	0.73
First-line treatment modality	Radiation vs. surgery	1.81	1.35	2.42	*<0*.*001*
	Systemic vs. surgery	1.17	0.99	1.39	0.06
	Multimodal vs. surgery	0.60	0.24	1.49	0.27
Histology	Chondrosarcoma vs. osteosarcoma	0.75	0.62	0.90	*0*.*002*
	Ewing's sarcoma vs. osteosarcoma	0.82	0.62	1.09	0.17
	Chordoma vs. osteosarcoma	0.27	0.10	0.73	*0*.*01*
	Other vs. osteosarcoma	0.75	0.59	0.96	*0*.*022*
Primary tumor site	Lower extremity vs. upper extremity	0.92	0.75	1.13	0.42
	Pelvis vs. upper extremity	1.58	1.26	1.99	*<0*.*001*
	Other vs. upper extremity	1.05	0.83	1.32	0.70
Tumor size	>8.0 cm vs. ≤8.0 cm	1.52	1.32	1.76	*<0*.*001*
Grade	Undifferentiated vs. poorly differentiated	1.07	0.94	1.23	0.31
Clinical staging	Stage II vs. stage I	0.96	0.77	1.20	0.72
	Stage III vs. stage I	0.97	0.69	1.38	0.87

TTI: time to treatment initiation; CI: confidence interval.

## Data Availability

The data used to support the findings of this study are included within the article.
